# Effects of Early-Childhood-Based Interventions Influencing Bones: A Systematic Review

**DOI:** 10.3390/jfmk9010002

**Published:** 2023-12-20

**Authors:** Markel Rico-González, Ricardo Martín-Moya, Adrián Moreno-Villanueva

**Affiliations:** 1 Department of Didactics of Musical, Plastic, and Corporal Expression, University of the Basque Country, UPV-EHU, 48940 Leioa, Spain; 2 Department of Physical Education and Sports, Faculty of Education and Sport Sciences, Campus of Melilla, University of Granada, 52006 Melilla, Spain; rmartinm@ugr.es; 3 Department of Health Sciences, University Isabel I of Castille, 09003 Burgos, Spain; more_adri@hotmail.com

**Keywords:** randomized controlled trials, health, teaching, education, physiology, physical education

## Abstract

A healthy lifestyle from early childhood is a crucial factor that influences bone-related factors in adulthood. In this context, physical education or psychomotricity from early childhood is an important opportunity to face this problem. The present article aims to systematically summarize school-based interventions, evaluated through randomized controlled trial design, that influence the bones of children from early childhood. A systematic review of relevant articles was carried out using four main databases (PubMed, ProQuest Central (including 26 databases), Scopus, and Web of Sciences) until 12 November 2023. From a total of 42 studies initially found, 12 were included in the qualitative synthesis. In brief terms, from early childhood and during puberty, children’s bones are particularly responsive to exercise, making this an ideal time for interventions to maximize bone health. Therefore, incorporating physical activity into school curriculums is a strategic approach for enhancing bone health in children. Mainly, plyometric exercises can significantly enhance bone density and geometry. Nevertheless, collaboration among educators, healthcare professionals, and parents is key for designing and implementing these effective interventions.

## 1. Introduction

Physical exercise is any bodily movement produced by skeletal muscles that expends energy, although different types of exercises may differ depending on the intensity, duration, and frequency of movements [1]. Examples are aerobic exercise (i.e., any activity that uses large muscle groups, can be maintained continuously, and is rhythmic in nature) [2] or force–velocity-based exercises (i.e., exercises where the maximal force is inversely related to the velocity of shortening) [3]. Further, the combination of different types of exercises may lead to different kinds of exercise such as weight-bearing aerobic exercises (i.e., impact activities or any other exercise in which the arms, feet, and legs are bearing the weight, such as walking, stair climbing, jogging, or dancing) or strength and/or resistance exercises (i.e., the joints are moved against some kind of resistance) [4].

For instance, the importance of physical exercise addressed by the United Nations Education, Scientific, and Cultural Organization (UNESCO) supports the importance of fueling an active lifestyle in all age ranges [5]. Unfortunately, current data highlight a sedentary lifestyle for 81% of children, forecasting a 15% relative reduction until 2030 [6]. This fact may lead to negative consequences for children’s health. An example to combat this is reducing the risk of suffering from non-communicable diseases in adulthood, such as high levels of obesity-related chronic diseases including diabetes, metabolic syndrome, or cardiovascular disease (e.g., hypertension, elevated cholesterol) [7,8,9,10]. Hence, different effects may be the reason for health risks and serious long-term consequences [11].

Among these long-term consequences are bone-related factors [12] such as bone mass, properties of constituent tissue, and bone geometry [13]. One of the most common diseases is osteoporosis, which is a musculoskeletal disease caused by a decrement in bone mass and destruction of the bone microstructure [14]. Osteoporosis affects 200 million people worldwide, resulting in 8.9 million fractures every year [15]. According to a recent systematic review and meta-analysis, the prevalence of osteoporosis is 18.3% globally (women = 23.1%; men = 11.7%) [16]. This disease is caused by changes in hormone secretion that lead to the breakdown of the original balance in the body and cause dysfunction in structures such as bones [17]. This disorder may be shown in the skeleton by a reduction in bone mineral density caused by fluctuations in osteoclast and osteoblast activity [14]. However, the problems that may affect bones may arise from early childhood. For example, some other diseases such as the metabolic bone disease of prematurity (characterized by skeletal demineralization or, in some cases, fragility fractures) arise from prenatal and postnatal factors, when an infant born preterm may be deprived of fetal mineral accumulation [18]. Fortunately, and considering the reduction in the quality of life and mobility that bone-related diseases provoke, many studies have highlighted non-pharmacological approaches for the prevention of osteoporosis regarding exercise [15], anthropometry, sleep disruptions (e.g., abnormal sleep duration, night shift work) [19], or nutrition (e.g., importance of calcium and vitamin D) [15], which may highlight the importance of caring for health-related habits from the first years of life.

In a study performed with participants from early childhood to 30 years of age [20], the authors highlighted that the peak bone mass occurs between the end of the second decade and early in the third decade of life, although it depends on the skeletal site. This idea supports the importance of the circumpubertal years for accruing bone minerals [20]. Thus, due to the importance of lifestyle in bone-related factors, the importance of practicing regular exercise from childhood for reducing the risk of osteoporosis and associated fractures has been highlighted. The convergence of this critical period of bone accrual and bone loading is considered a “window of opportunity” for the development of a healthy skeleton. This important period starts from early childhood, as a universal consensus highlights the effects of antecedents for reducing adult bone health problems [21]. This fact places education, in general, and physical education, in particular, as a crucial opportunity to face this problem.

To date, different systematic reviews have tried to analyze the effects of exercise on bone-related factors in children and adolescents. Examples are analyzing the influence of physical activity on bone strength in children and adolescents [21], the influence of sedentary behavior and bone health [12], and the comparison of the association between bone health and different intensities of accelerometer-derived habitual physical activity [20]. However, to the best of the authors’ knowledge, there is no systematic review that tried to analyze the effects of different school-based interventions, evaluated through randomized controlled trial design, on children’s bone-related factors from the early childhood period.

The present article aims to systematically summarize school-based interventions, evaluated through randomized controlled trial design, that influence the bones of children from early childhood.

## 2. Materials and Methods

### 2.1. Experimental Approach to the Problem

The present systematic review was carried out according to two guidelines: the Preferred Reporting Items for Systematic Reviews and Meta-Analyses (PRISMA) guidelines [22] and the guidelines for performing systematic reviews in sport sciences [23].

### 2.2. Information Sources

Four main databases were selected to identify the articles published before 12 November 2023: (1) PubMed, (2) ProQuest Central (including 26 databases), (3) Scopus, and (4) Web of Sciences.

### 2.3. Search Strategy

Following the PICO design provided by PRISMA, the following search strategy was used to look for relevant articles, where the authors were not blinded to journal names or manuscripts’ authors:

(preschool OR kindergarten OR “primary education” OR “elementary education” OR school) AND (“physical education”) AND (bone*) AND (“randomized controlled trial*”).

### 2.4. Inclusion/Exclusion Criteria

Two authors independently completed the search and compared results to ensure that the same articles were identified. Then, identifying information from the papers (title, authors, date, and database) was downloaded and transferred into an Excel spreadsheet (Microsoft Corporation, Redmond, WA, USA), and duplicates were removed. The remaining articles were independently screened for meeting inclusion and exclusion criteria (Table 1). Moreover, relevant articles not previously identified were also screened identically, and further studies that complied with the inclusion/exclusion criteria were included and labeled as “included from external sources”.

### 2.5. Data Extraction

Data extraction was prepared using an Excel spreadsheet in accordance with the Cochrane Consumers and Communication Review Group’s data extraction template (Group, 2016). The spreadsheet was used to assess inclusion and exclusion requirements for all selected studies. The process was independently conducted by the two authors. Any disagreement regarding study eligibility was resolved in a discussion. Full text articles that were excluded from the analysis were recorded with reasons for exclusion. All records were stored in the spreadsheet.

### 2.6. Assessment of Study Methodology

The Physiotherapy Evidence Database (PEDro) scale was used to assess the methodological quality of pre-test and post-test studies with experimental (EXP) and control (CON) groups randomly selected. The scale scores the internal study validity in a range of zero (low methodological quality) to 10 (high methodological quality). The score that each section receives can be from zero (“no”) to one (“yes”), depending on the quality obtained by each point. Ten items are measured in the scale. Studies that score from 9 to 10 on the PEDro scale are considered to be of excellent methodological quality. Studies with a score between six and eight have good methodological quality, between four and five, fair quality, and below four points, poor methodological quality [24].

## 3. Results

### 3.1. Identification and Selection of Studies

**Figure 1:**
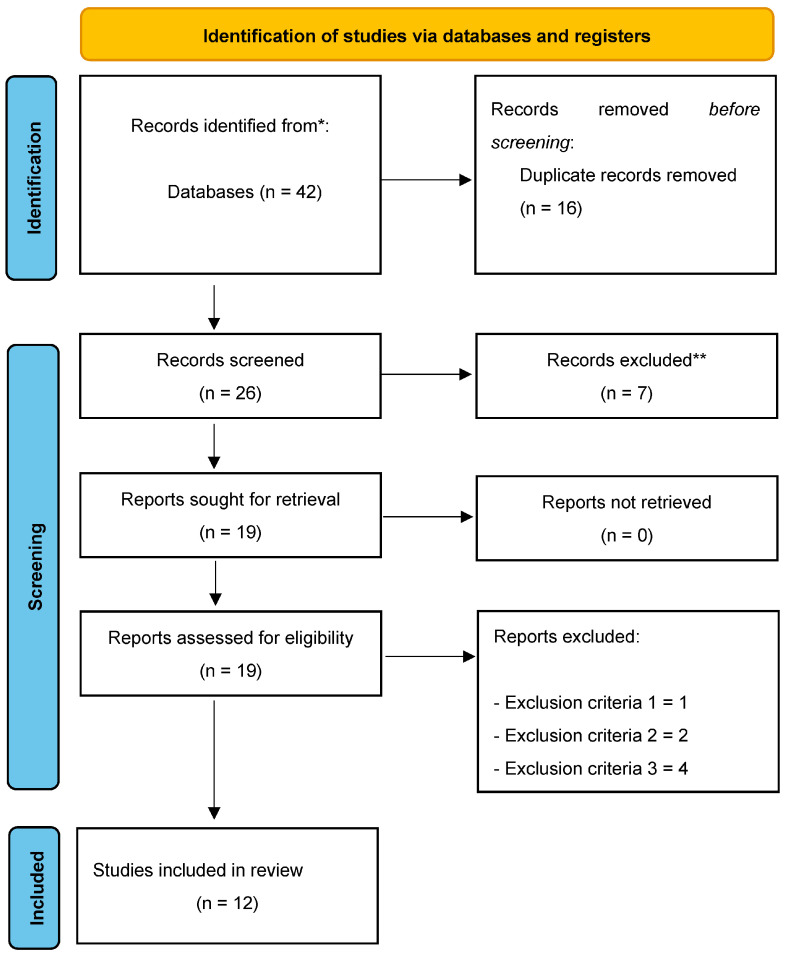
Flow diagram of the study.

A total of 42 original articles were found, of which 16 were duplicates. Thus, a total of 26 unique articles were identified. After checking titles and abstracts, seven articles were excluded because they did not meet inclusion criterion number five. The full text of the remaining 19 articles was then analyzed; one article was excluded because it did not meet inclusion criterion number one, two articles were excluded because they did not meet inclusion criterion number two, and four articles were excluded because they did not meet exclusion criterion number three. Thus, a total of 12 articles met all of the inclusion criteria and were included in the final qualitative synthesis (Figure 1).

### 3.2. Quality Assessment

The quality assessment for this systematic review can be found in Table 2:

From the 12 included articles, eight obtained an excellent methodological quality score [25,26,28,30,32,35,36], while the remaining four studies obtained a good methodological quality score [27,29,33,34]. None of the included studies had fair or poor methodological quality.

### 3.3. Study Characteristics

A total of 12 articles were finally selected. From them, only two dealt with subjects about pubertal age (13–17 years) [35,36], while the remaining 10 articles analyzed subjects in both prepubertal and early pubertal stages (6–12 years) [25,30,31,32,33,34]. In addition, it is worth noting the wide variety of countries that integrated this type of study, such as the USA [27,29], Switzerland [25,33,34], Israel [28], Canada [31,32], Denmark [30], and Australia [35,36].

Assessment tools: Dual-energy X-ray absorptiometry (DXA) was used [25,26,27,29,30,32,33,34,35,36], followed by ultrasound densitometer [28,35,36] and peripheral quantitative computed tomography (pQCT) [26,31].

Intervention protocols: Six articles carried out intervention programs based on plyometric exercises (mainly high-intensity training that involves explosive movements based on the Stretch-Shortening Cycle (SSC)). This cycle enhances the power of the subsequent movement [25,27,30,32,35,36], while the rest of the studies purposed other methodologies: the implementation of varied high-intensity activities in 10-min time periods [28,31,33,34] and sports practices [26,29,30].

Duration of the interventions: The duration of the different intervention protocols was characterized by heterogeneity, with periods ranging from 7 months [27,32] to 4 years [26].

Outcomes: Regarding the results for bone parameters, 10 out of 12 studies detected significant improvements in the intervention groups compared to their respective control groups [26,27,28,29,30,31,32,33,34,35].

Sex and age: Significant improvements in both sexes were found, although both agents achieved different magnitudes in the variables analyzed [26,28,31,32,35].

Finally, only two articles examined the adherence and continuity of the improvements acquired from the intervention programs after a few years [33,36], while only one highlighted the importance of physical activity habits during the three years without intervention [33].

The characteristics of the studies were extracted and are clustered in Table 3:

## 4. Discussion

The present article aimed to systematically summarize school-based interventions, evaluated through randomized controlled trial design, that influence the bones of children from early childhood.

In this review, there were only three programs developed during physical education classes. Therefore, since it is a strategic approach for enhancing bone health in children, taking advantage of the potential of this curricular subject and incorporating physical exercise programs in school curricula is necessary [37]. By targeting this intervention at an age where bones are most responsive to exercise, and ensuring that it is inclusive and adaptable to both genders, education from early childhood can play a pivotal role in establishing the foundation for lifelong bone health and overall well-being. Following MacKelvie et al. [32], early puberty may be a particularly suitable time for girls during growth for performing physical exercise interventions in order to have a positive effect on bone health. In this context, the onset of puberty brings an increment in girls’ estrogen levels, which significantly influence bone density. In fact, estrogen is vital for closing the growth plates and increasing the bone mineral density [38]. While the patterns of bone growth may vary between boys and girls, the benefits of physical activity for bone health are significant in both genders [39].

The regular plyometric exercises may also lead to an increment in bone mineral density in the lower extremities, which is a key factor in overall bone health [25,27,30,32,35,36]. Beyond density, the plyometric exercises can also improve bone geometry, making bones not just denser but also structurally sound [40]. Other types of exercise that can influence bones are weight-bearing physical activities such as running, jumping, and sports, which can stimulate bone growth through mechano-stimulation [41]. The stress placed on bones during these activities prompts bone-forming cells to increase bone mass and strength [42]. Following Larsen et al. [30], since peak bone mass is not yet achieved in this population group, this period is optimal for interventions like plyometric training to enhance bone properties. In addition, the plyometric exercises also contribute to improved muscular strength, coordination, and balance, which are important for overall physical development [43].

In this review, total body, femoral neck, and lumbar spine showed improvements in BMC and BMD during physical exercise programs in both sexes [27,32,33,34,35]. According to Lu et al. [44], the femoral neck, a critical region of the hip, benefits greatly from physical exercise. Given the femoral neck’s importance in weight-bearing and its vulnerability in later life (e.g., hip fractures), strengthening this area during childhood is particularly beneficial [37]. The lumbar spine is another key area that benefits from regular physical exercise. Exercises that involve bending, twisting, and lifting can increase the BMC and BMD in the vertebrae, which are vital for spinal health and posture [45]. Building strong bones during childhood can reduce the risk of osteoporosis and fractures later in life, especially in vulnerable areas like the hip and spine [46].

The impact of physical exercise programs on the distal radius and tibia properties in both boys and girls, with girls experiencing greater gains in the cortical area (CoA) and cortical thickness (CoTh) at the mid-tibia and radius [47], is an intriguing aspect of pediatric bone development and adaptation to exercise. These outcomes are highlighted in this systematic review in various studies [26,28]. The observation that girls show increments in CoA and CoTh in the mid-tibia and radius may be linked to hormonal differences and their response to mechanical loading during certain developmental stages [48]. Boys also benefit from exercise programs in terms of bone properties and the pattern of their bone development. Moreover, boys may respond differently to exercise due to factors like hormonal changes, growth rates, and the timing of growth spurts [49]. The cortical area and cortical thickness are key indicators of bone strength [50]. The cortical bone is the dense outer surface of bone that contributes significantly to bone strength and stability.

This review highlights the need to incorporate physical exercise programs into school curricula from early childhood for enhancing bone health in children, emphasizing that such interventions are particularly effective during early puberty in girls and beneficial for both genders, improving bone density, structure, and overall physical development. However, these findings should consider some limitations. One of them is the limited number of articles that were analyzed. It is important to raise awareness among educators, parents, or trainers of the importance of physical exercise in children to improve bone health. Another limitation would be the lack of studies carried out during the physical education classes during school hours.

## 5. Conclusions

Incorporating physical activity into school curriculums is a strategic approach to enhancing bone health in children. By targeting this intervention at an age where bones are most responsive to exercise, and ensuring it is inclusive and adaptable to both genders, schools can assume a pivotal role in establishing the foundation for lifelong bone health and overall well-being. The plyometric strength training offers a valuable method for improving lower extremity bone properties in prepubertal students. By capitalizing on the rapid bone development during this stage, the plyometric exercises can significantly enhance bone density and geometry. Collaboration among educators, healthcare professionals, and parents is a key for designing and implementing these effective interventions. For the aerobic and plyometric exercises, short rest periods are typical, especially if they are part of a game or play, while for structured strength training, 1–2 min of rest between sets is mainly recommended to prevent injuries. This consideration includes days with lighter or no scheduled exercise. Exercises should be suitable for the child’s age and physical development, knowing that younger children benefit more from play-like activities, while older children can engage in more structured exercises.

## Figures and Tables

**Table 1:** Inclusion/exclusion criteria.

Topic	Inclusion	Exclusion	Search Coherence
Population	Children from preschool, kindergartens, or elementary education	Children who do not attend preschool, kindergartens, or elementary education	preschool OR kindergarten OR “primary education” OR “elementary education” OR school
Intervention or exhibition	Children involved in school-aged intervention	Children not involved in preschools or elementary education	“physical education”
Results	Outcomes related to bones	Results extracted from teacher’s opinion, interviews, observations, perceptions, or experiences during a certain program. Program proposals without considering children in their studies. Study protocols.	Bone
Study design	Randomized controlled trials or parallel trials	Non-randomized controlled trials or parallel trials	“randomized controlled trial*”
Other criteria	Peer-reviewed, original, full-text studies	Articles written without peers, reviewing the complete original text studies.	-

**Table 2:** Methodological assessment of the included studies.

Reference	1	2	3	4	5	6	7	8	9	10	Score
Anliker et al. [25]	1	1	1	1	1	1	0	1	1	1	**9**
Daly et al. [26]	1	1	1	1	1	1	1	1	1	1	**10**
Fuchs et al. [27]	1	1	1	1	1	0	1	1	1	0	**8**
Goldstein et al. [28]	1	1	1	1	1	1	0	1	1	1	**9**
Gutin et al. [29]	1	1	0	1	1	1	0	1	1	0	**7**
Larsen et al. [30]	1	1	1	1	1	0	1	1	1	1	**9**
Macdonald et al. [31]	1	1	1	1	1	1	1	1	1	1	**10**
MacKelvie et al. [32]	1	1	1	1	1	1	0	1	1	1	**9**
Meyer et al. [33]	1	1	1	1	1	0	0	1	0	1	**7**
Meyer et al. [34]	1	1	0	1	1	1	0	1	1	1	**8**
Weeks et al. [35]	1	1	1	1	1	1	1	1	1	0	**9**
Weeks and Beck [36]	1	1	1	1	1	0	1	1	1	1	**9**

Note: Item 1 = subjects were randomly allocated to groups (in a crossover study, subjects were randomly allocated an order in which treatments were received); Item 2 = allocation was concealed; Item 3 = the groups were similar at baseline regarding the most important prognostic indicators; Item 4 = there was blinding of all subjects; Item 5 = there was blinding of all therapists who administered the therapy; Item 6 = there was blinding of all assessors who measured at least one key outcome; Item 7 = measures of at least one key outcome were obtained from more than 85% of the subjects initially allocated to groups; Item 8 = all subjects for whom outcome measures were available received the treatment or control condition as allocated or, where this was not the case, data for at least one key outcome was analyzed by “intention to treat”; Item 9 = the results of between-group statistical comparisons are reported for at least one key outcome; Item 10 = the study provides both point measures and measures of variability for at least one key outcome.

**Table 3:** Interventions for improving bone-related factors.

Reference	Aim	Sample	Intervention			Results			Conclusions
			Name/Groups	Intervention Main Ideas	Duration	Evaluation Tool	Variables	Results	
Anliker et al. [25]	Assess adaptations of the lower leg muscle-bone unit	Nº children: 45 Schools: 3 Country: Switzerland Mean age: 10.6 ± 1.1 years Range: 8–12 years	EXP (*n* = 22)	45’ sessions (×1/week) and 90’ sessions (×1/week) EXP: Jumping protocol (10 min) within physical education classes. CON: Education classes in accordance with the official curriculum by playing tag and/or related activities.	9 months	DXA XCT 3000 Scanner	FmILH Tibial bone strength/geometry	No significant changes in bone strength/geometry in EXP and CON (+3% to +1% points, respectively) Relationship between Fm1LH and BMC at the 14% site very strong in both groups (0.51 ≤ R2 ≤ 0.88)	In the children, growth and exercise did not increase bone strength proportionally, meaning that the adaptive processes were not tightly coupled and did not follow different time courses.
			CON (*n* = 23)						
Daly et al. [26]	Evaluate effects of a specialist-taught PE program on bone strength and body composition	Nº children: 727 Schools: 29 Country: 30 Mean age: 8.1 ± 0.3 years	EXP (*n* = 398) Lifestyle of Our Kids (LOOK) study	50’ sessions (×2/week) INT: Bluearth Foundation program CON: Current practice	4 years	DXA pQCT	BMC Radius and tibia (4% and 66% sites) bone structure Volumetric density	BMC similar in both groups. EXP girls had greater gains in cortical area (CoA) and cortical thickness (CoTh) at the mid-tibia (CoA = +7.5%; CoTh = +5.0%) and radius (CoA = +9.3%; CoTh = +14.4%) EXP boys had gains in mid-tibia CoTh (+5.2%)	Specialist-led school-based PE program improves cortical bone structure, due to reduced endocortical expansion.
			CON (*n* = 329)						
Fuchs et al. [27]	Investigate the effects of high-intensity jumping on hip and lumbar spine bone mass in children	Nº children: 89 Schools: 1 Country: USA Mean age: 7.5 ± 0.16	EXP (*n* = 45)	20’ sessions (×3/week) EXP: Jump to the 61-cm-high boxes, using the 20-cm-high boxes as a step CON: Non-impact/jumping exercises.	7 months	DXA	BMC Bone area BMD	Great changes in femoral neck (+4.5%) and lumbar spine (+3.1%) in EXP compared to CON. BMD at the lumbar spine significantly greater in EXP than in CON (+2.0%). Bone area had significantly greater increases in EXP at the femoral neck than controls (+2.9%)	Jumping at ground reaction forces of eight times body weight is a safe, effective, and simple method of improving bone mass at the hip and spine in children.
			CON (*n* = 44)						
Goldstein et al. [28]	Investigate the effect on young children who participated in a school-based intervention program on bone properties	Nº children: 295 Schools: 5 Country: Israel Range age: 6–8 years	EXP	EXP: 10’ weekly medium- to high-intensity activities CON: Current practice	1 year	Ultrasound Densitometer	Distal radius Tibia shaft	Distal radius properties improved significantly for both boys and girls in EXP (boys: +2.80%; girls: +3.30%) Tibia shaft properties only significantly improved for boys (+1.90%)	Distal radius properties of children can be positively affected by a short, easy to implement intervention program that does not require special resources.
			CON						
Gutin et al. [29]	Evaluate the effect of a 3-year after-school PA intervention on aerobic fitness and percent body fat	Nº children: 206 Schools: 18 Country: USA Mean age: 8.5 ± 0.6 years	EXP (*n* = 42) (FitKid program.)	80’ sessions (×5/week) EXP: Sport skills, aerobic fitness, strength, and flexibility + 40 min of vigorous PA CON: Current PA practice	3 years	DXA	BMD	EXP increased more than CON in BMD (+14.2%; *p* ≤ 0.01)	An after-school program focusing on MVPA had a beneficial effect on fitness and body composition, but this beneficial effect was lost during the summer.
			CON (*n* = 164)						
Larsen et al. [30]	Investigate whether musculoskeletal fitness of school children aged 8–10 years was affected by frequent intense PE sessions	Nº children: 295 Schools: 6 Country: Denmark Mean age: 9.3 ± 0.3 years Range age: 8–10 years	CST (*n* = 83) SSG (*n* = 96)	40’/session CST: ×4 sessions/week; 6–10 stations with plyometric and strength exercises SSG: ×5 sessions/week; 3 vs. 3 football or basketball games CON: ×5 sessions/week of current practice	10 months	DXA	aBMD BMC	SSG had higher change scores in leg aBMD compared with CON and CST (SSG vs. CON: 19 mg/cm^2^, 95% CI 11 to 39, *p* < 0.05; SSG vs. CST: 12 mg/cm^2^, 95% CI 3 to 21, *p* < 0.05). CST had higher change scores in whole-body BMC compared with CON (CST vs. CON: 25 g, 95% CI 10 to 39, *p* < 0.05)	Well-organized intense physical education classes can contribute positively to develop musculoskeletal health in young children.
			CON (*n* = 116)						
Macdonald et al. [31]	Determine whether “AS! BC” program would improve tibial bone strength in boys and girls	Nº children: 410 Schools: 10 Country: Canada (Mean age: 10.3 ± 0.6 years)	EXP (*n* = 281) (Action Schools! BC program)	40’ sessions (×2/week) EXP: 1º component: +15’ PA (×5 days/week) 2º component: ×3 periods of 3’ PA (×4 days/week) CON: Current practice (40’ sessions; ×2/week)	16 months	pQCT	BSI SSIp	EXP boys had a greater increase in BSI (+774.6 mg^2^/mm^4^; 95% CI: 672.7, 876.4) than CON boys (+650.9 mg^2^/mm^4^; 95% CI: 496.4, 805.4) EXP boys had a greater increase in SSIp (+198.6 mm^3^; 95% CI: 182.9, 214.3) than CON boys (+177.1 mm^3^; 95% CI: 153.5, 200.7) Change in BSI and SSIp was similar between CON and EXP girls	A simple, pragmatic program of daily activity enhances bone strength at the distal tibia in prepubertal boys.
			CON (*n* = 129)						
MacKelvie et al. [32]	Evaluate the effects of PE intervention program on bone mineral accrual in prepubertal and early pubertal girls	Nº children: 87 Schools: 14 Country: Canada Mean age: 10 ± 0.7 years Range age: 8.7–11.7 years	EXP (*n* = 45)	EXP: 10’ sessions (×3/week) circuit of varied jumping activities CON: 10’ sessions of stretching warm-up at the beginning of their PE classes	7 months	DXA	BMC and BMD of: Lumbar spine Proximal femur Femoral neck	No difference in change in bone parameters between prepubertal EXP and CON EXP early pubertal girls gained more bone at the femoral neck and lumbar spine (1.5% to 3.1%) than CON early pubertal girls (*p* < 0.05)	In girls, early puberty may be a particularly opportune time during growth for simple exercise interventions to have a positive effect on bone health.
			CON (*n* = 42)						
Meyer et al. [33]	Measured BMC and aBMD 3 years after cessation of the KISS intervention to investigate whether the beneficial short-term effects persisted	Nº children: 502 Schools: 15 Country: Switzerland Mean age: 9.2 ± 2.2 years Range age: 6–12 years	EXP (*n* = 297) (KISS program)	45’ sessions (×3/week) EXP: Additional 10’ Impact loading activities (×2/week) CON: Current PA practice.	9 months	DXA	BMC and aBMD of: Total body Femoral neck Total hip Lumbar spine	EXP showed significantly higher Z-scores of BMC at total body (0.157 units (0.031–0.283); *p* = 0.015), femoral neck (0.205 units (0.007–0.402); *p* = 0.042) and at total hip (0.195 units (0.036 to 0.353); *p* = 0.016) compared to CON. EXP had higher Z-scores of aBMD for total body (0.167 units (0.016 to 0.317); *p* = 0.030) compared to CON.	Beneficial effects on BMC of a nine month KISS program appeared to persist over three years. Part of the maintained effects may be explained by current physical activity habits.
			CON (*n* = 205)						
Meyer et al. [34]	Determine whether a school-based PA program during one school-year influences BMC and BMD	Nº children: 502 Schools: 15 Country: Switzerland Mean age: 9.2 ± 2.2 years Range age: 6–12 years	EXP (*n* = 297) (KISS program)	45’ sessions (×3/week) EXP: Additional 10’ Impact loading activities (×2/week) CON: Current PA practice	9 months	DXA	BMC and aBMD of: Total body Femoral neck Total hip Lumbar spine	Compared to CON, EXP children showed statistically significant increases in BMC of total body, femoral neck, and lumbar spine by 5.5%, 5.4% and 4.7% (all *p* < 0.05), respectively. EXP children had greater increases in BMD of total body and lumbar spine by 8.4% and 7.3% (both *p* < 0.01), respectively, compared to CON.	A general school-based PA intervention can increase bone health in elementary school children of both genders, particularly before puberty.
			CON (*n* = 205)						
Weeks et al. [35]	Determine the effect of POWER PE program on parameters of bone and muscle strength in healthy adolescent boys and girls	Nº children: 81 Schools: 1 Country: Australia Mean age: 13.8 ± 0.4 years Range age: 12–14 years	EXP (*n* = 43) (POWER PE program)	(×2/week) EXP: 10’ Jumping exercises at the beginning of each PE class. CON: Usual PE warm-up activities	8 months	DXA Ultrasound Densitometer	BMC Bone geometric	EXP boys experienced more improvements in calcaneal broadband ultrasound attenuation (+5.0%) compared to CON (+1.4%) EXP girls had more improved femoral neck BMC (+13.9%) and lumbar spine BMAD (+5.2%) than CON (+4.9% and +1.5%, respectively)	The POWER PE program improved indices of bone in healthy adolescent boys and girls in the high school PE setting without the need for additional staffing or equipment.
			CON (*n* = 38)						
Weeks & Beck [36]	Determine if the musculoskeletal benefits of a POWER PE program in healthy adolescent boys and girls were maintained 3 years later	Nº children: 29 Schools: 1 Country: Australia Mean age: 17.3 ± 0.4 years Range age: 16–18 years	EXP (*n* = 11) (POWER PE program)	(×2/week) EXP: 10’ Jumping exercises at the beginning of each PE class. CON: Usual PE warm-up activities	3 years later	DXA Ultrasound Densitometer	BMC Bone geometric	No significant group differences in three-year change in broadband ultrasound attenuation or BMC at any site (*p* > 0.05)	Osteogenic benefits of an 8-month in-school jumping intervention for adolescents can be sustained for at least three years (into young adulthood).
			CON (*n* = 18)						

Note: BMC = Bone mineral content; BMD = Bone mineral density; BSI = Bone strength index; CI = Confidence interval; CON = Control group; CST = Circuit strength training; DXA = Dual-energy X-ray absorptiometry; EXP = Experimental group; FmILH = Multiple one-legged hopping; PA = Physical activity; PE = Physical exercise; pQCT = Peripheral quantitative computed tomography; SSG = Small sided game; SSIp = Polar strength strain index.
